# Complementary iTRAQ-based proteomic and RNA sequencing-based transcriptomic analyses reveal a complex network regulating pomegranate (*Punica granatum* L.) fruit peel colour

**DOI:** 10.1038/s41598-018-30088-3

**Published:** 2018-08-17

**Authors:** Xiang Luo, Da Cao, Haoxian Li, Diguang Zhao, Hui Xue, Juan Niu, Lina Chen, Fuhong Zhang, Shangyin Cao

**Affiliations:** Zhengzhou Fruit Research Institute, Chinese Academy of Agricultural Sciences, Zhengzhou, 450009 P. R. China

## Abstract

Peel colour is an important factor affecting the marketability of pomegranate fruits. Therefore, elucidating the genetic mechanism of fruit peel colour development may be useful for breeding pomegranate cultivars with enhanced fruit peel colours. In this study, we combined an iTRAQ-based proteome-level analysis with an RNA sequencing-based transcriptome-level analysis to detect the proteins and genes related to fruit peel colour development in pomegranate. We analysed the ‘Tunisia’ (red fruit) and ‘White’ (white fruit) pomegranate cultivars at two stages of fruit development. A total of 27 differentially abundant proteins (increased abundance) and 54 differentially expressed genes (16 up-regulated and 38 down-regulated) were identified from our proteomics and transcriptomics data. The identified proteins and genes contribute to pomegranate fruit peel colour by participating in the biosynthesis of anthocyanins, stilbenoids, diarylheptanoids, gingerols, flavonoids, and phenylpropanoids. Several candidate proteins and genes corresponded to enzymes related to general reactions (PAL, 4CL, DFR, LDOX/ANS, CHS, and F3′5′H) and glycosylation (GT1 and UGAT) of compounds and pigments related to the colour of pomegranate fruit peel. Complementary proteome- and transcriptome-level analyses revealed a complex molecular network controlling fruit peel colour. The candidate genes identified in this study may be useful for the marker-based breeding of new pomegranate cultivars.

## Introduction

Pomegranate originated in Central Asia, including Iran, Afghanistan, and Caucasia, and is one of the oldest cultivated fruits. Pomegranate trees were introduced into China approximately 2000 years ago^1^, and their fruits are valued for their beauty and their desirable flavour, colour, and health benefits^2^. The peel colour of pomegranate fruits is a major factor affecting consumer acceptance and marketability. Thus, one of the main objectives of pomegranate breeders is to enhance fruit peel colour.

The biological and genetic factors regulating fruit peel colour have been studied extensively. A previous study revealed that storage temperature is the main factor affecting longkong fruit peel colour and physiology^3^. Light can also affect fruit colour, for example, light stimulates the synthesis of carotenoids in citrus fruit peel, resulting in the accumulation of β-cryptoxanthin during a specific stage of fruit development^4^. Changes in the carotenoid content of citrus fruit peel during fruit-colouring stages are significantly correlated with variations in peel colour^5^. In grape, a lack of light accelerates chlorophyll breakdown and induces carotenoid accumulation, resulting in intensely coloured fruits^6^. The development of peach fruit colour could be enhanced by bagging fruits in white non-woven polypropylene bags^7^. Researchers have also shown that light-responsive transcriptional regulatory factors regulate the anthocyanin levels in bagged Chinese sand pears^8^. In apple, light induces the expression of a MYB transcription factor gene associated with anthocyanin biosynthesis, and its expression level is related to the final fruit skin colour^9^. However, the pear MYB transcription factor gene *PcMYB10* (an ortholog of *MdMYBa*/*MdMYB10*, which controls the pigmentation of apple fruit skin) is not directly responsible for mediating fruit colour^9^. Thus, fruit peel colour is a complex trait that involves physiological, biochemical, and molecular processes. At present, little is known about the development of pomegranate fruit peel colour.

High-throughput technologies for measuring gene expression levels and protein abundance have enabled transcriptome- and proteome-level analyses of developmental processes, gene functions, adaptations, and physiological stress responses in plants^10^. The genes related to the biosynthesis of natural products in the pomegranate fruit peel have been investigated based on a *de novo* transcriptome set^11^, but a comprehensive examination of the encoded proteins has not been conducted. The isobaric tags for relative and absolute quantitation (iTRAQ) method represents a powerful option for analysing actively produced proteins. This method allows for the simultaneous identification and quantification of proteins from multiple samples based on isotope labelling combined with multidimensional liquid chromatography and tandem mass spectrometry (LC-MS/MS)^12^. Because of its high sensitivity and accuracy, the iTRAQ method has been used to elucidate post-transcriptional regulatory activities in mango^13^, oriental melon^14^, strawberry^15^, and peach^16^. We recently sequenced and assembled the pomegranate genome (unpublished data). In the present study, we combined iTRAQ-based proteomics and RNA sequencing-based transcriptomics analyses to identify the proteins and genes related to fruit peel colour during two fruit development stages in two pomegranate cultivars, ‘Tunisia’ and ‘White’. Then, the differentially abundant proteins (DAPs) and differentially expressed genes (DEGs) were subjected to Gene Ontology (GO) and Kyoto Encyclopedia of Genes and Genomes (KEGG) pathway enrichment analyses. Candidate genes related to fruit peel colour were predicted based on genome annotation and metabolic pathway information. In this study, we comprehensively characterised the development of pomegranate fruit peel colour at the proteome and transcriptome levels. The identified candidate genes may be useful for molecular marker-assisted pomegranate breeding.

## Results

### Identification of DAPs using iTRAQ technology

**Figure 1:**
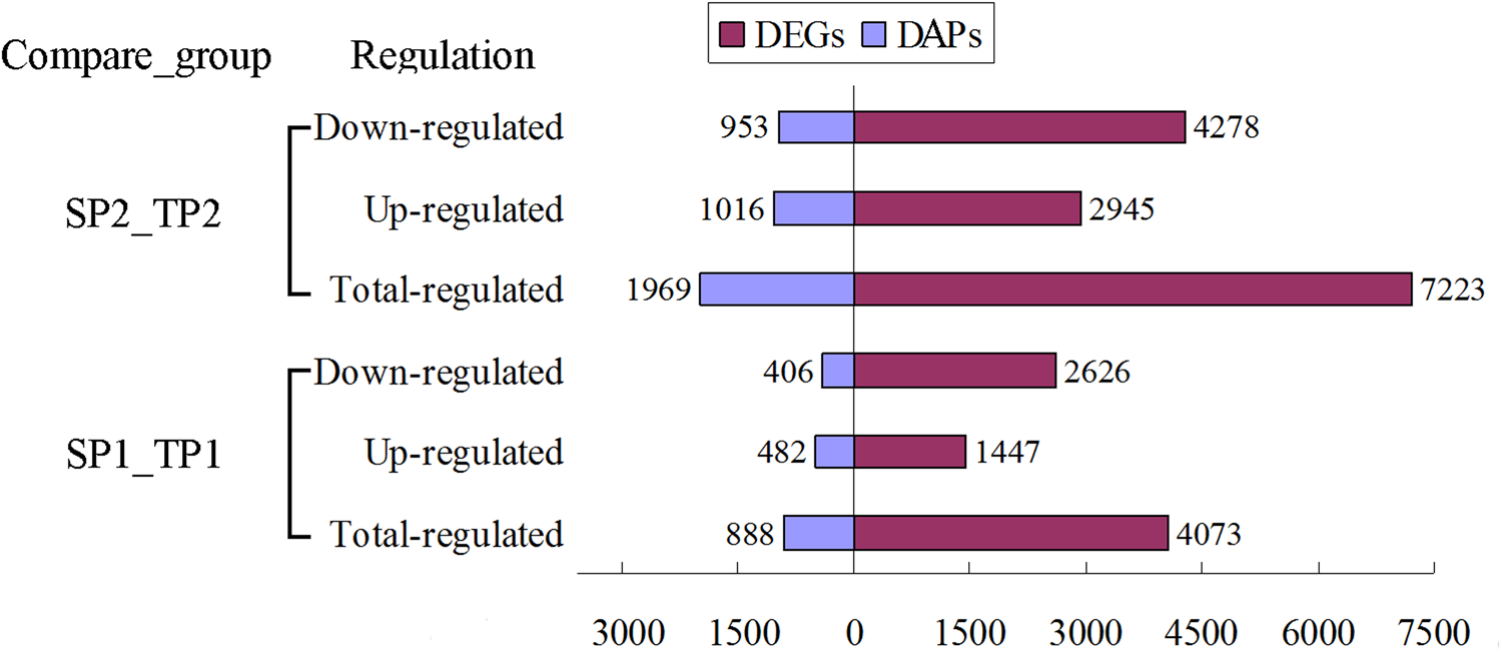
Differentially expressed genes and differentially abundant proteins in pomegranate fruit peels identified in different comparisons. SP1_TP1: comparison between ‘White’ and ‘Tunisia’ fruit peels during fruit colouring stage. SP2_TP2: comparison between ‘White’ and ‘Tunisia’ fruit peels during fruit ripening period.

The DAPs in two groups during the fruit colouring (SP1_TP1) and ripening (SP2_TP2) stages were identified and quantified using iTRAQ and LC-MS/MS analysis. Accordingly, 237,421 spectra were generated, and 18,407 unique peptides and 5,357 proteins were identified with a false discovery rate (FDR) of ≤1% (Supplementary Table S1). Among these proteins, there were 888 and 1,969 DAPs in SP1_TP1 and SP2_TP2, respectively, with a fold-change >1.2 (mean value of all compared groups) or <0.85 and a *P* value (*t*-test of all comparison groups) of <0.05 (Fig. 1 and Table 1). In the comparison between SP1 and TP1 (SP1_TP1), 482 DAPs were more abundant and 406 DAPs were less abundant in TP1 than in SP1. Similarly, 1,016 DAPs were more abundant and 953 DAPs were less abundant in TP2 than in SP2. There were nearly twice as many DAPs in SP2_TP2 than in SP1_TP1. These results indicated that many proteins showed differential abundance between the two fruit development stages, with more DAPs at the second analysed stage.

**Table 1:** Summary of proteins and transcripts detected from iTRAQ and RNA sequencing data.

	Proteins		mRNAs	
	SP1_TP1	SP2_TP2	SP1_TP1	SP2_TP2
Unique proteins\genes detected	5357	5357	18918	20545
Significantly DAPs\DEGs	888	1969	4073	7223
Up-regulated	482	1016	1447	2945
Down-regulated	406	953	2626	4278
Shared proteins/genes	552		1684	
Shared proteins/genes (up-regulated)	311	304	655	636
Shared proteins/genes (down-regulated)	241	248	1029	1048

### Transcriptional analysis of DEGs

To verify transcriptional-level changes, four cDNA libraries (i.e., SP1, SP2, TP1, and TP2) were constructed using total RNA extracted from the fruit peels of the two pomegranate cultivars. A total of 64.85, 61.99, 59.99, and 67.46 million raw sequence reads were generated from the SP1, SP2, TP1, and TP2 libraries, respectively (Supplementary Table S1). After removing low-quality reads and adaptor sequences, 37.43, 35.63, 34.68, and 38.76 million clean reads were obtained for SP1, SP2, TP1, and TP2, respectively, with 45.23%, 44.06%, 42.25%, and 41.89% of the reads mapped to the reference genome, respectively. The proportion of unique reads that could be aligned to the genome sequence ranged from 33.33% for TP2 to 37.34% for SP1. Finally, 18,918 and 20,545 unique genes were detected in SP1_TP1 and SP2_TP2, respectively. Compared with the SP group, the TP group had 4,073 (1,447 up-regulated and 2,626 down-regulated) significant DEGs (i.e., |log2(TP/SP)| > 1 and *P* ≤ 0.001) at the fruit colouring stage, and 7,223 (2,945 up-regulated and 4,278 down-regulated) significant DEGs at the ripening stage (Fig. 1 and Table 1). These results suggested that many genes were differentially expressed between the two fruit development stages in two pomegranate cultivars, with more DEGs at the fruit ripening stage.

### Comparative analysis between protein abundance and gene expression levels

**Figure 2:**
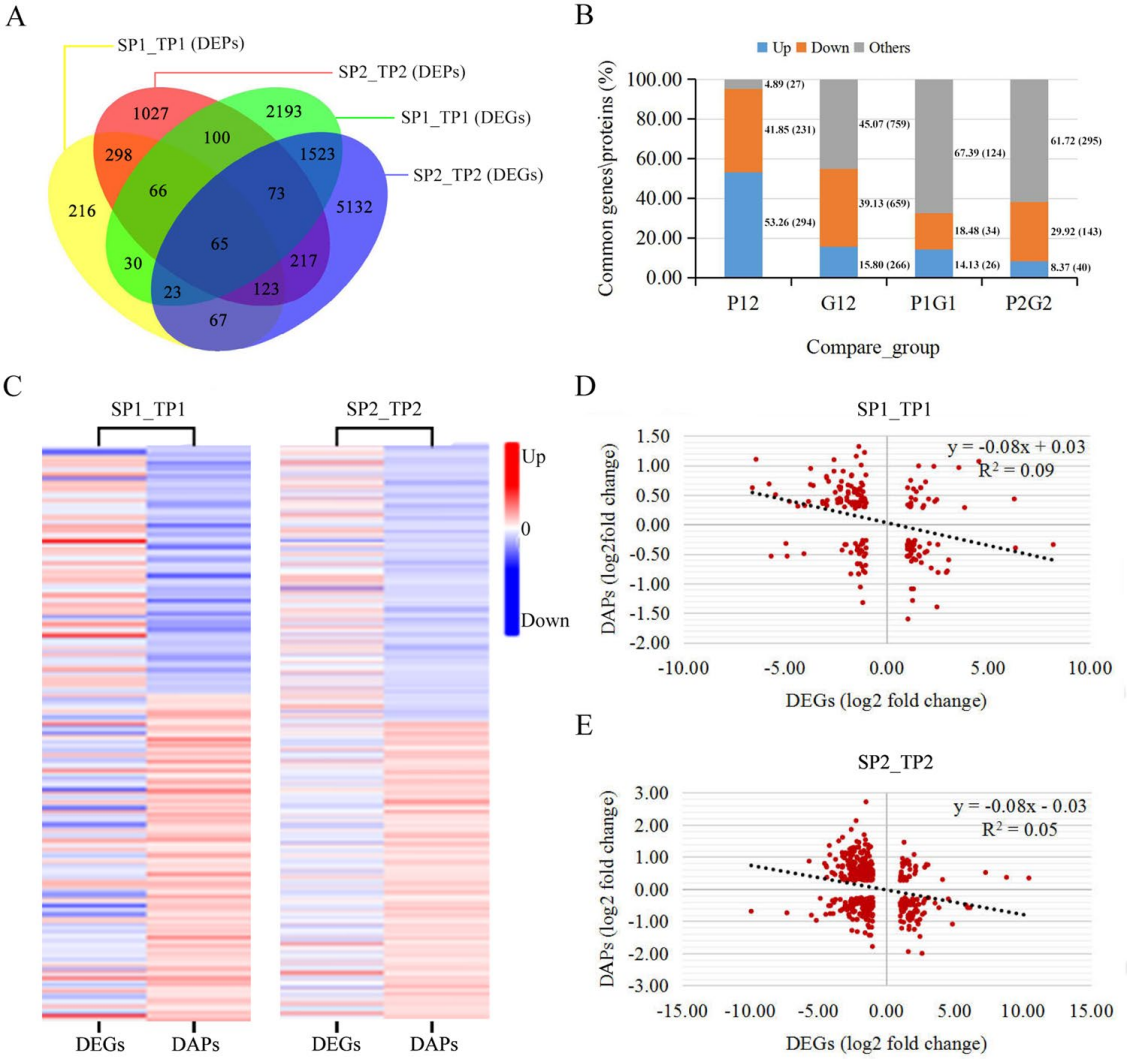
Protein abundance and gene expression levels in different comparisons. (**A**) Venn diagram of proteins and genes. (**B**) Changes in the common DAPs and DEGs between groups. P12: common DAPs between SP1_TP1 and SP2_TP2; G12: common DEGs between SP1_TP1 and SP2_TP2; P1G1: SP1_TP1 genes in which associated transcript level and protein abundance exhibited opposite trends; P2G2: SP2_TP2 genes in which associated transcript level and protein abundance exhibited opposite trends. (**C**) Comparison of changes in transcript and protein levels of DEGs and DAPs. (**D**) Correlations between transcript levels and protein abundance of DEGs and DAPs in SP1_TP1. (**E**) Correlations between transcript levels and protein abundance of DEGs and DAPs in SP2_TP2.

To evaluate the relationship between transcript levels and protein abundance, we compared the DAPs and DEGs between the two analysed fruit development stages. A total of 552 shared DAPs and 1,684 shared DEGs were identified during the comparison between SP1_TP1 and SP2_TP2 (Fig. 2A and Table 1). Among the shared DAPs, 311 were more abundant and 241 were less abundant in SP1_TP1 than in SP2_TP2, while 304 were more abundant and 248 were less abundant in SP2_TP2 than in SP1_TP1 (Fig. 2B). Of the common DEGs, 655 were up-regulated and 1,029 were down-regulated in SP1_TP1, whereas 636 were up-regulated and 1,048 were down-regulated in SP2_TP2 (Fig. 2B). Furthermore, 184 and 478 DAPs and their corresponding DEGs were identified in SP1_TP1 and SP2_TP2, respectively (Fig. 2C). Of these, only 60 DAPs (26 with increased abundance and 34 with decreased abundance) and 183 DAPs (40 with increased abundance and 143 with decreased abundance) were regulated in the same direction as their corresponding DEGs in SP1_TP1 and SP2_TP2, respectively (Fig. 2B,C). There were more DEGs than DAPs in both SP1_TP1 and SP2_TP2, with considerable differences between the trends in transcript levels and the trends in protein abundance. To analyse the consistency between transcriptomic and proteomic changes during the development of fruit peel colour, we conducted a correlation analysis using the quantitative data for DAPs and DEGs (Fig. 2D,E; Supplementary Table S2). Pearson’s correlation tests indicated that the fold-changes in the DAPs were significantly (*P* < 0.001) negatively correlated with the fold-changes in the corresponding DEGs (*r*_pearson_ = −0.29 and −0.23 in SP1_TP1 and SP2_TP2, respectively). Thus, there was a poor correlation between transcript levels and protein abundance.

### KEGG pathway annotations for DAPs and DEGs

**Figure 3:**
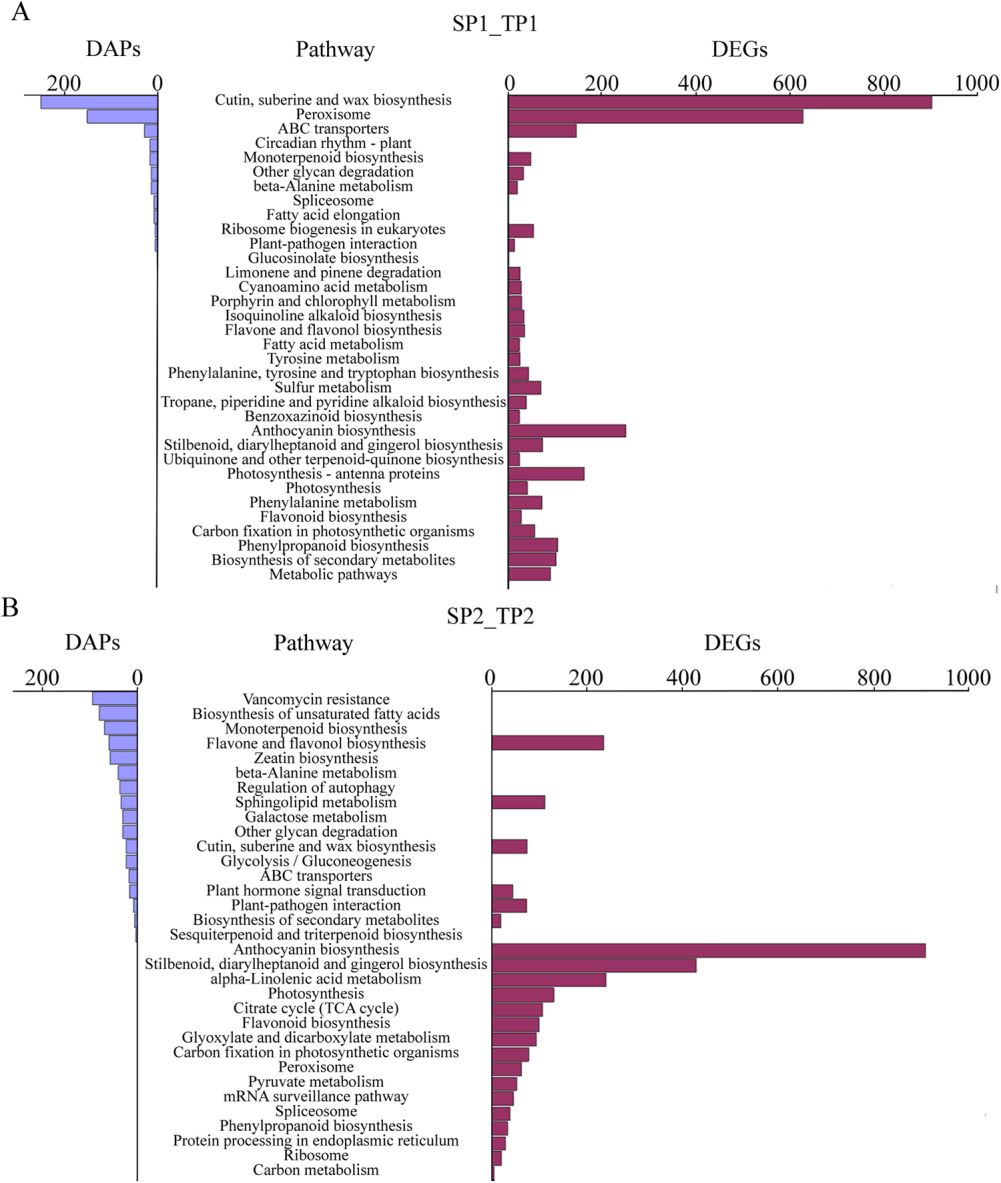
KEGG pathways associated with differentially expressed genes and differentially abundant proteins in (**A**) SP1_TP1 and (**B**) SP2_TP2. Number of proteins (left) and number of genes (right) associated with a pathway are indicated.

To annotate the functions of genes and proteins, we conducted a pathway enrichment analysis of the DAPs and DEGs in SP1_TP1 and SP2_TP2 based on the KEGG database (Fig. 3). In SP1_TP1, 521 DAPs were significantly enriched in 11 pathways (corrected *P* value < 0.05; Fig. 3A), while 3,181 DEGs were significantly enriched in 30 pathways (corrected *P* value < 0.05; Fig. 3B). The following pathways were enriched with both DAPs and DEGs in SP1_TP1: anthocyanin biosynthesis; stilbenoid, diarylheptanoid, and gingerol biosynthesis; photosynthesis; phenylalanine metabolism; flavonoid biosynthesis; phenylpropanoid biosynthesis; biosynthesis of secondary metabolites; and metabolic pathways. In SP2_TP2, 630 DAPs were significantly enriched in 14 pathways, while 2,936 DEGs were significantly enriched in 22 pathways (corrected *P* value < 0.05; Fig. 3). The following pathways were common to both the DAPs and DEGs in SP2_TP2: anthocyanin biosynthesis; stilbenoid, diarylheptanoid, and gingerol biosynthesis; photosynthesis; flavonoid biosynthesis; α-linolenic acid metabolism; and peroxisome and phenylpropanoid biosynthesis. An additional comparative analysis indicated that four pathways (i.e., anthocyanin biosynthesis; stilbenoid, diarylheptanoid, and gingerol biosynthesis; flavonoid biosynthesis; and phenylpropanoid biosynthesis) were common between SP1_TP1 and SP2_TP2. Thus, these four pathways were further investigated as candidate pathways related to the development of pomegranate fruit peel colour.

### Identification of DAPs and DEGs associated with candidate pathways

**Figure 4:**
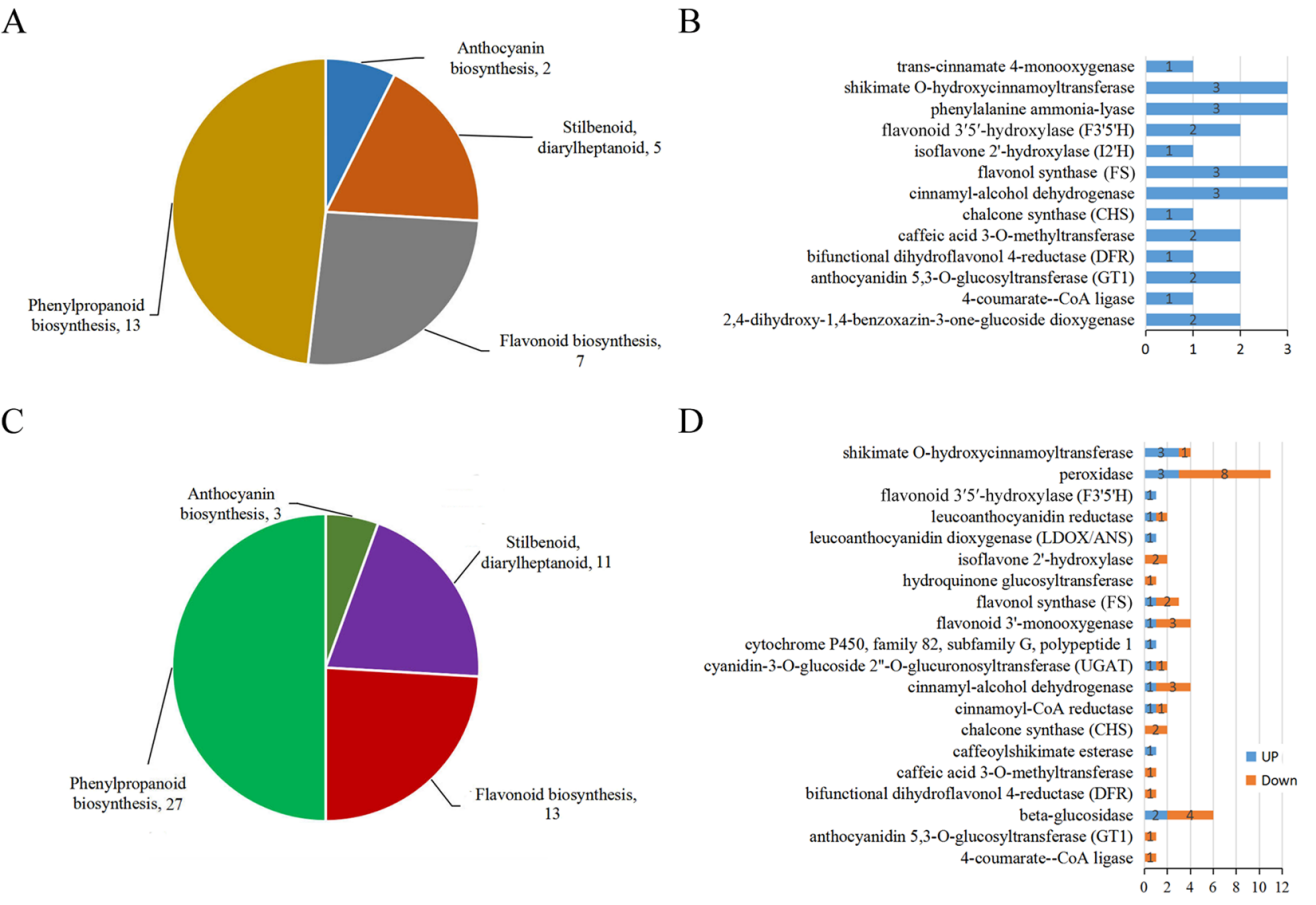
Summary of number of (**A**) differentially abundant proteins (DAPs) and (**C**) differentially expressed genes (DEGs) in each pathway. (**B**) Annotation and abundance changes of DAPs. (**D**) Annotation and expression level changes of DEGs. Numbers represent number of genes and proteins.

A total of 51 and 89 DAPs detected in SP1_TP1 and SP2_TP2, respectively, were associated with the four candidate pathways, with 34 DAPs common to both sample groups (Supplementary Table S3). Additionally, 241 and 397 DEGs identified in SP1_TP1 and SP2_TP2, respectively, were related to the four candidate pathways, including 100 DEGs common to both sample groups (Supplementary Table S4). After filtering out the DAPs and DEGs that were differentially regulated between SP1_TP1 and SP2_TP2, 27 DAPs with increasing abundance and 54 DEGs (16 up-regulated DEGs and 38 down-regulated DEGs) were annotated as being involved in anthocyanin biosynthesis; stilbenoid, diarylheptanoid, and gingerol biosynthesis; flavonoid biosynthesis; and phenylpropanoid biosynthesis (Fig. 4A,C; Tables 2 and 3). These DAPs and DEGs likely influence pomegranate fruit peel colour at the proteome and transcriptome levels. Except for Gglean022299.1, the DAPs represented 13 crucial enzymes (Fig. 4B). With the exception of *Gglean025205*.*1*, *Gglean018889*.*1*, and *Gglean015246*.*1*, the DEGs encoded 20 pivotal enzymes (Fig. 4D). Nine enzymes were affected by corresponding genes with differentially regulated trends at the transcriptome level. More DEGs than DAPs were involved in the formation of pomegranate fruit peel colour, and the DEGs showed complex differential regulation at the transcriptome level. Most of the genes encoding the DAPs were not included among the DEGs, consistent with the observed differences between the proteome and transcriptome data (Fig. 2B). Only four of the DEGs [i.e., two flavonoid biosynthesis genes (*Gglean009951*.*1* and *Gglean020650*.*1*) and two phenylpropanoid biosynthesis genes (*Gglean015001*.*1* and *Gglean015003*.*1*)] encoded DAPs. Further analyses revealed that the abundance of most of the flavonoid and phenylpropanoid biosynthesis enzymes increased but the expression levels of their corresponding genes decreased during fruit colour development.

**Table 2:** Pathways associated with candidate differentially abundant proteins (DAPs) between ‘Tunisia’ and ‘White’ pomegranate cultivars in TP1_SP1 and TP2_SP2 comparisons.

Pathway	Common DAP	Regulation	KO id	Annotation [EC no.]	Fold (TP1/SP1)	*P* value	Fold (TP2/SP2)	*P* value
Anthocyanin biosynthesis	Gglean022014.1	Up	K12938	anthocyanidin 5,3-O-glucosyltransferase (GT1) [EC:2.4.1.-]	1.46	0.03	1.38	0.00
	Gglean030887.1	Up	K08237		1.59	0.05	1.36	0.00
Stilbenoid, diarylheptanoid biosynthesis	Gglean022299.1	Up	K00517	—	1.51	0.01	1.77	0.00
	Gglean017162.1	Up	K13260	isoflavone 2′-hydroxylase (I2^,^H) [EC:1.14.13.89]	1.54	0.00	1.90	0.02
	Gglean020115.1	Up	K13065	shikimate O-hydroxycinnamoyltransferase [EC:2.3.1.133]	1.58	0.01	1.95	0.02
	Gglean017092.1	Up			1.74	0.01	2.46	0.00
	Gglean030413.1	Up	K00487	trans-cinnamate 4-monooxygenase [EC:1.14.13.11]	1.63	0.01	1.31	0.00
Flavonoid biosynthesis	Gglean024451.1	Up	K13082	bifunctional dihydroflavonol 4-reductase (DFR) [EC:1.1.1.219]	1.86	0.04	1.96	0.00
	Gglean009951.1	Up	K00660	chalcone synthase (CHS) [EC:2.3.1.74]	2.23	0.01	2.32	0.00
	Gglean021885.1	Up	K05278	flavonol synthase (FS) [EC:1.14.11.23]	1.57	0.00	1.76	0.03
	Gglean002595.1	Up			1.21	0.04	1.40	0.02
	Gglean002270.1	Up			1.61	0.03	2.12	0.00
	Gglean024933.1	Up	K00475	flavonoid 3′5′-hydroxylase (F35H) [EC:1.14.11.9]	2.08	0.01	2.41	0.00
	Gglean020650.1	Up			2.33	0.00	2.16	0.00
Phenylpropanoid biosynthesis	Gglean004667.1	Up	K13229	2,4-dihydroxy-1,4-benzoxazin-3-one-glucoside dioxygenase [EC:1.14.20.2]	1.64	0.01	1.42	0.03
	Gglean005438.1	Up			1.31	0.01	1.34	0.00
	Gglean008923.1	Up	K01904	4-coumarate–CoA ligase [EC:6.2.1.12]	1.24	0.01	2.22	0.00
	Gglean004749.1	Up	K01188	beta-glucosidase [EC:3.2.1.21]	1.25	0.01	1.30	0.01
	Gglean030344.1	Up	K13066	caffeic acid 3-O-methyltransferase [EC:2.1.1.68]	1.51	0.00	3.00	0.00
	Gglean015152.1	Up			1.44	0.01	2.20	0.00
	Gglean015001.1	Up	K00083	cinnamyl-alcohol dehydrogenase [EC:1.1.1.195]	1.46	0.01	1.78	0.00
	Gglean015223.1	Up			1.26	0.01	1.29	0.03
	Gglean015003.1	Up			1.52	0.03	1.42	0.01
	Gglean023090.1	Up	K10775	phenylalanine ammonia-lyase [EC:4.3.1.24]	1.60	0.02	1.37	0.00
	Gglean026319.1	Up			1.98	0.01	1.41	0.00
	Gglean025624.1	Up			2.48	0.01	1.23	0.01
	Gglean017092.1	Up	K13065	shikimate O-hydroxycinnamoyltransferase [EC:2.3.1.133]	1.74	0.01	1.73	0.00

**Table 3:** Pathways associated with candidate differentially expressed genes (DEGs) between ‘Tunisia’ and ‘White’ pomegranate cultivars in TP1_SP1 and TP2_SP2 comparisons.

pathway	Common DEGs	Regulation	KO id	Annotation [EC no.]	log2(TP1/SP1)	*P* value	log2(TP2/SP2)	*P* value
Anthocyanin biosynthesis	*Gglean030299*.*1*	Down	K12938	anthocyanidin 5,3-O-glucosyltransferase (GT1) [EC:2.4.1.-]	−1.39	0.00	−1.54	0.00
	*Gglean024784*.*1*	Down	K18823	cyanidin-3-O-glucoside 2′′-O-glucuronosyltransferase (UGAT) [EC:2.4.1.254]	−3.84	0.00	−1.92	0.00
	*Gglean024783*.*1*	Up			1.20	0.00	1.66	0.00
Stilbenoid, diarylheptanoid biosynthesis	*Gglean025205*.*1*	Down	—	—	−6.44	0.00	−2.28	0.00
	*Gglean018889*.*1*	Up	—	—	4.07	0.00	1.37	0.00
	*Gglean000381*.*1*	Up	K17961	cytochrome P450, family 82, subfamily G, polypeptide 1 [EC:1.14.-.-]	5.09	0.00	6.11	0.00
	*Gglean026629*.*1*	Up	K05280	flavonoid 3′-monooxygenase [EC:1.14.13.21]	1.32	0.00	1.64	0.00
	*Gglean019885*.*1*	Down	K13260	isoflavone 2′-hydroxylase [EC:1.14.13.89]	−3.02	0.00	−2.66	0.00
	*Gglean019884*.*1*	Down			−2.71	0.00	−2.18	0.00
	*Gglean015246*.*1*	Down	—	—	−2.53	0.00	−1.81	0.00
	*Gglean015686*.*1*	Down	K13065	shikimate O-hydroxycinnamoyltransferase [EC:2.3.1.133]	−3.22	0.00	−1.77	0.00
	*Gglean020272*.*1*	Up			1.96	0.00	2.92	0.00
	*Gglean004433*.*1*	Up			1.78	0.00	1.87	0.00
	*Gglean013382*.*1*	Up			1.19	0.00	1.07	0.00
Flavonoid biosynthesis	*Gglean024449*.*1*	Down	K13082	bifunctional dihydroflavonol 4-reductase (DFR) [EC:1.1.1.219]	−1.10	0.00	−3.98	0.00
	*Gglean009951*.*1*	Down	K00660	chalcone synthase (CHS) [EC:2.3.1.74]	−1.50	0.00	−3.11	0.00
	*Gglean012297*.*1*	Down			−1.25	0.00	−1.56	0.00
	*Gglean025015*.*1*	Down	K05280	flavonoid 3′-monooxygenase [EC:1.14.13.21]	−2.64	0.00	−3.69	0.00
	*Gglean027001*.*1*	Down			−2.66	0.00	−1.77	0.00
	*Gglean000166*.*1*	Down			−2.59	0.00	−1.32	0.00
	*Gglean001689*.*1*	Up	K05278	flavonol synthase (FS) [EC:1.14.11.23]	1.49	0.00	2.12	0.00
	*Gglean002078*.*1*	Down			−2.69	0.00	−2.19	0.00
	*Gglean018019*.*1*	Down			−1.06	0.00	−1.11	0.00
	*Gglean026620*.*1*	Up	K05277	leucoanthocyanidin dioxygenase (LDOX/ANS) [EC:1.14.11.19]	2.57	0.00	4.83	0.00
	*Gglean024550*.*1*	Up	K13081	leucoanthocyanidin reductase [EC:1.17.1.3]	1.10	0.00	1.94	0.00
	*Gglean002570*.*1*	Down			−3.16	0.00	−1.02	0.00
	*Gglean020650*.*1*	Down	K00475	flavonoid 3′5′-hydroxylase (F35H) [EC:1.14.11.9]	−1.09	0.00	−3.18	0.00
Phenylpropanoid biosynthesis	*Gglean004943*.*1*	Down	K01904	4-coumarate–CoA ligase [EC:6.2.1.12]	−1.37	0.00	−2.12	0.00
	*Gglean004745*.*1*	Up	K01188	beta-glucosidase [EC:3.2.1.21]	1.33	0.00	5.81	0.00
	*Gglean014679*.*1*	Down			−1.53	0.00	−2.84	0.00
	*Gglean025214*.*1*	Down			−7.85	0.00	−2.79	0.00
	*Gglean026132*.*1*	Down			−1.07	0.00	−2.50	0.00
	*Gglean028143*.*1*	Down			−2.66	0.00	−1.88	0.00
	*Gglean023909*.*1*	Up			3.22	0.00	2.82	0.00
	*Gglean015153*.*1*	Down	K13066	caffeic acid 3-O-methyltransferase [EC:2.1.1.68]	−2.14	0.00	−1.06	0.00
	*Gglean028708*.*1*	Up	K18368	caffeoylshikimate esterase [EC:3.1.1.-]	1.33	0.00	1.61	0.00
	*Gglean008959*.*1*	Down	K09753	cinnamoyl-CoA reductase [EC:1.2.1.44]	−1.39	0.00	−2.07	0.00
	*Gglean003647*.*1*	Up			1.84	0.00	1.81	0.00
	*Gglean015001*.*1*	Down	K00083	cinnamyl-alcohol dehydrogenase [EC:1.1.1.195]	−1.60	0.00	−3.79	0.00
	*Gglean015003*.*1*	Down			−1.59	0.00	−4.39	0.00
	*Gglean002744*.*1*	Down			−1.20	0.00	−1.26	0.00
	*Gglean017886*.*1*	Up			3.65	0.00	1.87	0.00
	*Gglean031042*.*1*	Down	K08237	hydroquinone glucosyltransferase [EC:2.4.1.218]	−1.17	0.00	−2.32	0.00
	*Gglean018745*.*1*	Down	K00430	peroxidase [EC:1.11.1.7]	−1.15	0.00	−2.60	0.00
	*Gglean004996*.*1*	Up			1.61	0.00	1.14	0.00
	*Gglean004579*.*1*	Down			−3.24	0.00	−2.24	0.00
	*Gglean027772*.*1*	Down			−1.58	0.00	−3.38	0.00
	*Gglean022575*.*1*	Down			−1.12	0.00	−1.02	0.00
	*Gglean018674*.*1*	Down			−1.11	0.00	−3.38	0.00
	*Gglean007149*.*1*	Down			−1.75	0.00	−1.84	0.00
	*Gglean016128*.*1*	Down			−2.31	0.00	−2.94	0.00
	*Gglean019365*.*1*	Up			1.83	0.00	1.87	0.00
	*Gglean022649*.*1*	Up			5.02	0.00	4.12	0.00
	*Gglean029864*.*1*	Down			−1.01	0.00	−1.77	0.00

### Pathway analysis of candidate DAPs and DEGs

**Figure 5:**
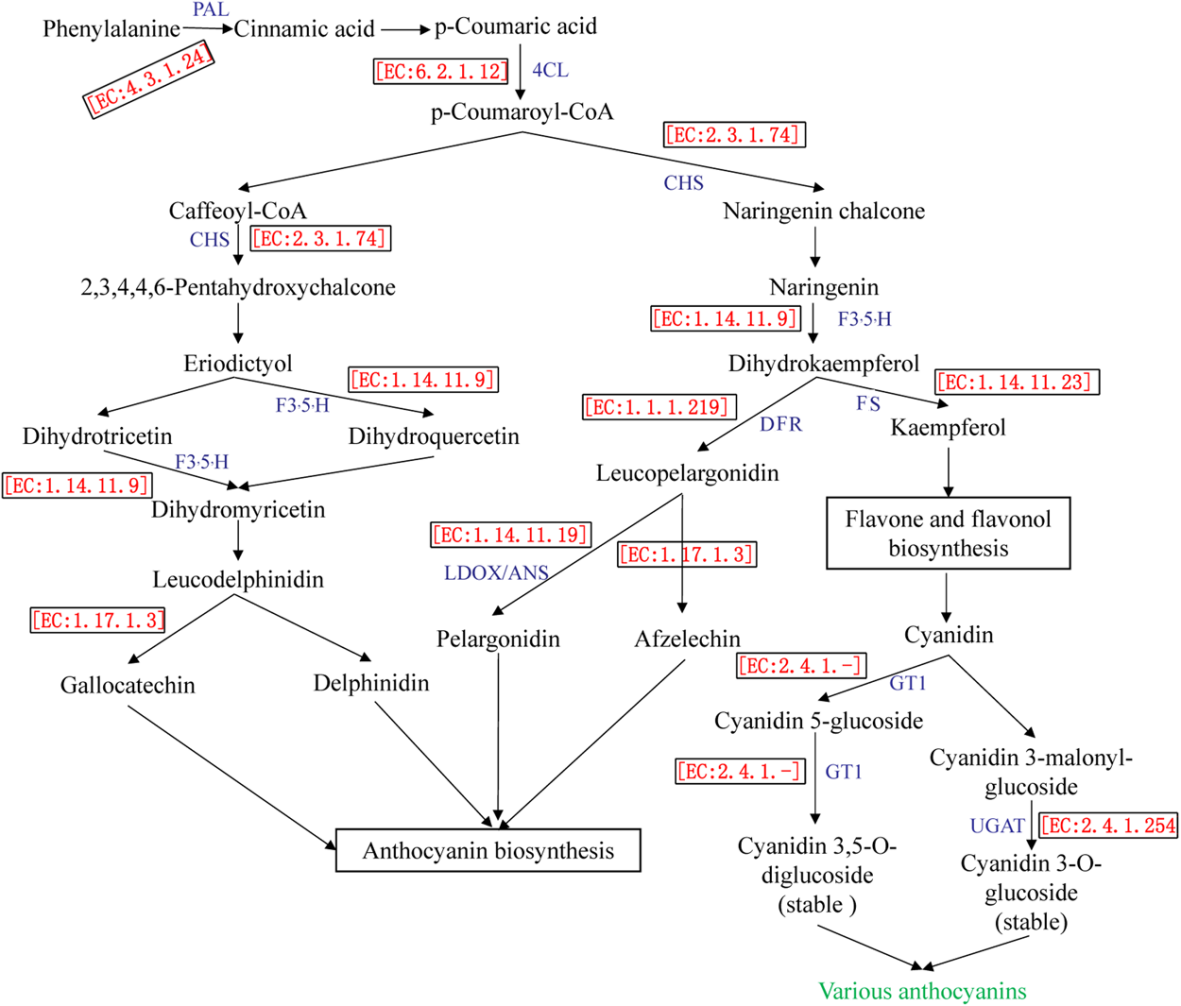
Differentially abundant proteins (DAPs) and differentially expressed genes (DEGs) related to anthocyanin biosynthesis in pomegranate fruit peels. Numbers and abbreviations of key enzymes are shown in red and blue text, respectively.

A pathway analysis was completed to characterise the functions of the candidate DAPs and DEGs more comprehensively. A total of 13 DAPs and 17 DEGs corresponded to enzymes involved in anthocyanin biosynthesis (Fig. 5), including phenylalanine ammonia-lyase (PAL, three proteins), 4-coumarate-CoA ligase (4CL, one protein and one transcript), dihydroflavonol 4-reductase (DFR, one protein), leucoanthocyanidin dioxygenase/anthocyanidin synthase (LDOX/ANS, one transcript), chalcone synthase (CHS, one protein and two transcripts), flavanone 3′,5′ hydroxylase (F3′5′H, two proteins and one transcript), [EC:1.1.1.234] (one transcript), [EC:1.14.11.23] (three proteins and three transcripts), [EC:1.14.13.21] (three transcripts), [EC:1.17.1.3] (two transcripts), GT1 (two proteins and one transcript), and UGAT (two transcripts). These genes and proteins represent the core enzymes in anthocyanin biosynthesis, which influence pomegranate fruit peel colour development. The DEGs *Gglean009951*.*1* and *Gglean020650*.*1* encoded the DAPs CHS and F3′5′H, respectively, while the DEGs *Gglean015001*.*1* and *Gglean015003*.*1* both encoded DAP cinnamyl-alcohol dehydrogenase (CAD) [EC:1.1.1.195] (Supplementary Fig. S1), which is involved in phenylpropanoid biosynthesis.

### Validation of gene expression levels

The expression levels of 12 genes, including *Gglean009951*.*1*, *Gglean020650*.*1*, *Gglean015001*.*1*, and *Gglean015003*.*1*, were determined by quantitative real-time polymerase chain reaction (qRT-PCR). The expression patterns were similar to those generated from high-throughput sequencing (Supplementary Fig. S2). The trends in the expression levels of *Gglean009951*.*1*, *Gglean020650*.*1*, *Gglean015001*.*1*, and *Gglean015003*.*1* revealed by qRT-PCR were inconsistent with the changes in the abundance of their encoded proteins as detected in the iTRAQ analysis. These results suggested that deep sequencing represents an accurate and efficient method for analysing pomegranate gene expression levels and the abundance of their corresponding proteins.

## Discussion

Peel colour is a key factor influencing the marketability of fruits. Thus, elucidating the genetic mechanism of the regulation of fruit peel colour may be useful for breeders interested in generating new pomegranate cultivars with enhanced fruit colours. Consequently, fruit peel development has been a topic of interest among fruit crop researchers and has been extensively studied. To date, however, no quantitative trait loci related to fruit colour have been identified, and none of the genes related to pomegranate fruit peel colour have been confirmed.

**Figure 6:**
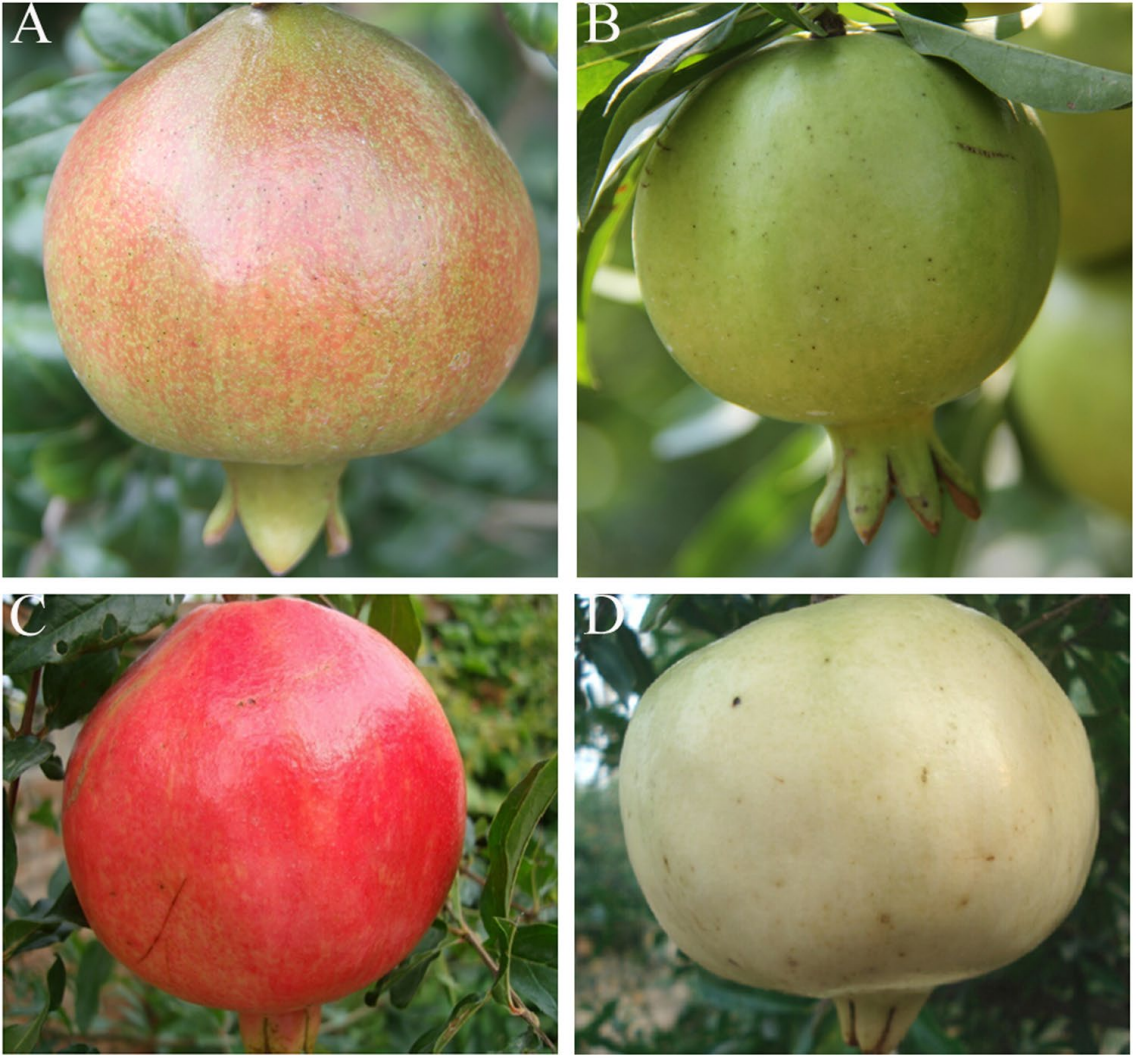
Comparison of fruits from two pomegranate cultivars during fruit colouring and ripening stages. (**A**) ‘Tunisia’ and (**B**) ‘White’ fruits during fruit colouring stage. (**C**) ‘Tunisia’ and (**D**) ‘White’ fruits during fruit ripening stage.

In this study, more DAPs and DEGs were detected between the ‘Tunisia’ and ‘White’ cultivars at the fruit ripening stage than at the fruit colouring stage, suggesting that greater changes in peel colour occurred at the ripening stage (Fig. 6). An integrated quantitative proteomics and transcriptomics analysis revealed that there were more DEGs than DAPs in both SP1_TP1 and SP2_TP2, with only a few of the DEGs encoding the DAPs. These results were similar to those reported for potato^17^ and orchid^18^. There were inconsistencies between the trends in transcript levels and the trends in protein abundance. These results may indicate that protein abundance is affected by post-translational modifications and splicing events in cells, rather than changes in gene transcript levels^19^. A correlation analysis revealed a negative correlation between protein abundance and the expression levels of the corresponding genes (Fig. 2D,E). An earlier study also reported a relatively poor correlation between transcriptome and proteome data (*r*_pearson_ = 0.27–0.40)^20^. A possible explanation for the low correlation between transcript levels and protein abundance is that the transcription level can fluctuate more quickly than protein translation and modification processes. Thus, changes in the abundance of a protein occur after the level of its corresponding transcript has stabilised^18^. Our results suggest that iTRAQ and transcriptomic analyses are complementary methods for profiling candidate proteins mediating specific physiological processes, including pomegranate fruit peel colour development.

A KEGG pathway enrichment analysis was completed to avoid false positive results, and the common pathways shared by DAPs and DEGs were selected for further study. Accordingly, we functionally annotated 27 proteins and 54 genes from among the thousands of detected DAPs and DEGs. These proteins and genes were associated with 13 and 20 critical enzymes, respectively, which contributed to pomegranate fruit peel colour mainly by catalysing reactions in pathways related to anthocyanin biosynthesis; stilbenoid, diarylheptanoid, and gingerol biosynthesis; flavonoid biosynthesis; and phenylpropanoid biosynthesis (Fig. 4; Tables 2 and 3). A total of 13 DAPs and 17 DEGs were detected as core regulators of anthocyanin biosynthesis in pomegranate fruit peel (Fig. 5), implying that the colour differences between white and red peels are due to variable anthocyanin production^21^.

Anthocyanin biosynthesis is fairly complex, and is associated with diverse metabolites including phenylpropanoids and flavonoids^22^. To date, diverse genes (i.e., *PAL*, *4CL*, *DFR*, *LDOX/ANS*, *CHS*, and *F3′5′H*) involved in the regulation of anthocyanin biosynthesis have been identified and their functions in the formation of pigments have been characterised in fruit crops including Rosaceae species^23^, apple^24^, peach^25^, and pear^8^. In the current study, the expression levels of genes encoding F3′5′H and LDOX/ANS were up-regulated and the abundance of their corresponding proteins also increased during fruit colour development (Fig. 4; Tables 2 and 3). In contrast, although the abundance of PAL, 4CL, CHS, and DFR proteins increased, their corresponding genes were down-regulated during fruit colour development (Fig. 4; Tables 2 and 3). Both PAL and 4CL are critical for the conversion of phenylalanine to anthocyanins^13^. A decrease in transcription but an increase in the abundance of the encoded proteins ensured a sufficient supply of anthocyanin precursors (e.g., p-coumaroyl-CoA) in ‘Tunisia’ fruit peels (Fig. 5). Similarly, CHS catalyses the conversion of p-coumaroyl-CoA to naringenin chalcone (Fig. 5). This represents a key regulatory step during the synthesis of flavonoids, which is a major pigment in many flowers, leaves, and fruits^26,27^. Naringenin is converted into several anthocyanin-related substances (e.g., dihydrokaempferol, leucopelargonidin, and pelargonidin) by the actions of F3′5′H, DFR, and LDOX/ANS (Fig. 5). These enzymatic reactions have been documented in sweet cherry^28^, apple^24^, mango^13^, and peach^25^. The results of a previous study indicated that a single nucleotide polymorphism in *PgLDOX* is responsible for the ‘white’ anthocyanin-less phenotype of pomegranate fruits^21^. Thus, we hypothesised that these genes may be conserved and play a crucial role in the anthocyanin biosynthesis related to pomegranate fruit peel colour development.

Caffeoyl-CoA, CHS, and F3′5′H catalyse a chain of reactions to generate dihydromyricetin, a coloured chemical compound related to leucoanthocyanidins^29^. Leucoanthocyanidin reductase [EC:1.17.1.3] converts leucoanthocyanidin to gallocatechin as well as eucopelargonidin to afzelechin (Fig. 5). These two reactions ultimately produce anthocyanins. It is possible that these pathways also contribute to pomegranate fruit peel colour development.

Plant scientists have long been interested in flavonoid pigments because they are responsible for the diverse colours (e.g., yellow, red, purple, and blue) of various organs, including fruits and flowers^30,31^. In this study, changes in *FS* transcript levels and the abundance of FS protein tended to induce the biosynthesis of flavones and flavonols (Fig. 5). Down- and up-regulated expression of *Gglean024784*.*1* and *Gglean024783*.*1*, respectively, may help regulate the UGAT-catalysed conversion of cyanidin 3-malonylglucoside to the stable compound cyanidin 3,5-O-diglucoside. Additionally, GT1 can catalyse the glycosylation of the 5-OH and the 3-OH groups on the anthocyanidin molecule. Although the *GT1* transcript levels decreased during fruit colour development, the abundance of GT1 protein increased, which likely promoted the conversion of cyanidin to cyanidin 5-glucoside and then to cyanidin 3,5-O-diglucoside. These reactions result in the production of various anthocyanins (Fig. 5). This is consistent with the findings of a previous study on anthocyanin biosynthesis in rose^32^. Our results also confirmed that the glucosyltransferase-catalysed glycosylation at two different positions on the precursor molecule is not limited to rose species^32^. Thus, we believe that genes regulating anthocyanin biosynthesis *via* glycosylation play important roles in the stabilization of pigments in pomegranate fruit peels.

In addition to anthocyanins and flavonoids, other secondary metabolites are produced in the phenylpropanoid biosynthesis pathway by the activities of enzymes such as CAD [EC:1.1.1.195]. We detected three CAD proteins with increased abundance and four *CAD* genes among the DEGs (one up-regulated and three down-regulated), including *Gglean015001*.*1* and *Gglean015003*.*1*, during fruit colour development in pomegranate. An earlier study confirmed that CAD belongs to the polyphenol oxidase (PPO) family^33^, and catalyses the interconversion between aldehydes and alcohols (Supplementary Fig. S1). Disrupting the expression of *CAD* genes will result in the atypical incorporation of hydroxycinnamaldehyde into lignin, thereby modifying the cell wall structure^34^. A previous study revealed that PPO protects red pear fruit or leaf cells against pathogens by catalysing the synthesis of lignin and quinines^35^. Thus, CAD may induce the synthesis of lignin and quinines to alter the cell wall structure to enhance pathogen resistance, which may explain the relatively strong disease resistance of red pomegranate fruits^36^.

The regulatory functions of the other identified enzymes in pathways related to anthocyanin biosynthesis, especially those in the stilbenoid, diarylheptanoid, and gingerol biosynthesis pathways, have not been characterised. Combined analyses of metabolomics data may further clarify their functions in relation to the regulation of pomegranate fruit peel development. In conclusion, the transcriptome and proteome data generated in this study revealed a complex transcriptional and translational network regulating pomegranate fruit peel colour development. These candidate proteins and genes may be useful for marker-based breeding of new pomegranate cultivars.

## Methods

### Plant materials

The ‘Tunisia’ and ‘White’ pomegranate cultivars analysed in this study were grown and managed according to local production practices in Xingyang, China. Both cultivars were obtained from the Zhengzhou Fruit Research Institute, Chinese Academy of Agricultural Sciences, Zhengzhou, China. The blooming stage was defined as the period during which 50% of the pomegranate flowers were open. According to our observations, the fruit development periods of the two cultivars were similar. The differences in the fruit peel colours between the two cultivars developed during the fruit colouring stage (60 days after blooming) and especially during the ripening stage (120 days after blooming). The peel of ‘Tunisia’ fruits reddened during the fruit colouring and ripening stages, in contrast to the white fruits of the ‘White’ cultivar (Fig. 6). During the fruit colouring and ripening periods, the peels of three normally growing fruits were removed and pooled. Three replicates were collected. The peels were immediately frozen in liquid nitrogen for 30 min and stored at −80 °C until use. For convenience, the ‘Tunisia’ and ‘White’ fruit peels were abbreviated as TP and SP, respectively.

Phenotypic variation, correlation, and linear regression analyses were completed using SPSS (version 19.0) (IBM Corp., Armonk, NY, USA).

### Protein extraction

Proteins were extracted from two biological replicates of pomegranate fruit peels as previously described^37^. Briefly, Lysis Buffer 3 [8 M urea and 40 mM Tris-HCl containing 1 mM PMSF (phenylmethylsulfonyl fluoride), 2 mM EDTA (ethylenediaminetetraacetic acid), and 10 mM DTT (dithiothreitol), pH 8.5] was added to fruit peel samples, which were then ground. After centrifuging the ground material at 25,000 × g for 20 min at 4 °C, five volumes of 10% TCA/acetone with 10 mM DTT were added to the supernatant to precipitate proteins at −20 °C. The precipitation step was repeated with acetone alone until the supernatant became colourless. The precipitated proteins were air-dried and resuspended in Lysis Buffer 3. Samples were sonicated on ice for 5 min to resuspend proteins and then centrifuged as before. The resulting supernatants were incubated at 56 °C with 10 mM DTT for 1 h to reduce the proteins. The proteins were then alkylated with 55 mM iodoacetamide at room temperature for 45 min in darkness. Five volumes of acetone were added to samples to precipitate proteins at −20 °C. Proteins were resuspended in Lysis Buffer 3 and then sonicated on ice for 5 min. The protein concentration was determined by Bradford’s method using bovine serum albumin (BSA) as the standard.

### Protein digestion and iTRAQ labelling

Protein solutions (100 µg) were diluted 4-times with 100 mM tetraethylammonium bromide, after which proteins were digested overnight with Trypsin Gold (Promega, Madison, WI, USA) at 37 °C (40:1, protein:trypsin). The peptides were desalted with a Strata X C18 column (Phenomenex, Torrance, CA, USA) and then vacuum-dried according to the protocol recommended by the manufacturer. Peptide samples were labelled using iTRAQ 8-plex kits (AB Sciex Inc., MA, USA). The two ‘Tunisia’ samples (TP1 and TP2) were labelled with iTRAQ tags 113 and 115, while the two ‘White’ samples (SP1 and SP2) were labelled with tags 117 and 119.

### Peptide fractionation

Peptides were separated using a Shimadzu LC-20AB HPLC Pump system coupled with a high-pH RP column. The peptides were first reconstituted in Buffer A (5% ACN and 95% H_2_O, with the pH adjusted to 9.8 with ammonia) to a final volume of 2 ml, which was then loaded onto a column containing 5-μm particles (Phenomenex). Peptides were separated with a gradient of 5% Buffer B (5% H_2_O and 95% ACN, with the pH adjusted to 9.8 with ammonia) for 10 min, 5–35% Buffer B for 40 min, and 35–95% Buffer B for 1 min at a flow rate of 1 ml/min. The system was maintained in 95% Buffer B for 3 min, followed by a decrease to 5% Buffer B within 1 min and equilibration with 5% Buffer B for 10 min. The eluants were monitored by measuring the absorbance at 214 nm, and fractions were collected every 1 min. The eluted peptides were pooled as 20 fractions and vacuum-dried.

### Analysis by LC-MS/MS

Each fraction was resuspended in Buffer A (2% ACN and 0.1% FA in water) and centrifuged at 20,000 × g for 10 min. The supernatant was loaded onto a C18 trap column (5 μl/min for 8 min) on an LC-20AD nano-HPLC instrument equipped with an autosampler (Shimadzu, Kyoto, Japan). The peptides were eluted from the column and separated using an analytical C18 column (75-μm inner diameter) packed in-house. The 8%–35% Buffer B (2% H_2_O and 0.1% FA in ACN) gradient was applied at 300 nl/min over 35 minutes, and then increased to 60% in 5 min, maintained at 80% for 5 min, and then decreased to 5% over 0.1 min. The column was equilibrated with 5% Buffer B for 10 min. The eluted peptides underwent nanoelectrospray ionisation before being analysed by MS/MS (Q-Exactive mass spectrometer; Thermo Fisher Scientific, San Jose, CA, USA) coupled with the HPLC system.

### Protein identification and quantification based on iTRAQ data

Proteins were identified and quantified using the Mascot 2.3.02 search engine (Matrix Science, Boston, MA, USA). Our genome database was used to identify proteins with the IQuant program^38^. To assess the confidence of the peptide identifications, the PSMs were pre-filtered at a PSM-level FDR of 1%. The identified peptide sequences were then assembled into a set of accurately identified proteins based on the “simple principle”. To quantify proteins, peptides were automatically selected by calculating the reporter peak area using the default parameters of the Mascot software package. The resulting data set was auto-bias corrected, and the variations resulting from the unequal mixing of samples with different labels were eliminated. The DAPs between the TP and SP samples were defined as the proteins with a fold-change of >1.2 (mean value of all compared groups) or <0.85 and a *P* value (*t*-test of all comparison groups) of <0.05. To minimise the protein-level false-positive rates, a protein FDR of 1%, which was based on an established “picked” protein FDR strategy^39^, was estimated after proteins were tentatively identified (protein-level FDR ≤ 0.01).

### Nuclear RNA extraction and sequencing

Total RNA was extracted from the fruit peels of the two pomegranate cultivars sampled at two fruit development stages for subsequent RNA sequencing analysis. The RNA was extracted from two biological replicates of frozen samples (100 mg) using the RNAprep Pure Plant Kit (Tiangen Biotech, Beijing, China). An ND 1000 spectrophotometer (NanoDrop Technologies, Wilmington, DE, USA) was used to evaluate the quality of the extracted RNA. Additionally, RNA with an RNA Integrity Number > 8 according to the 2100 Bioanalyzer (Agilent, USA) was used to prepare cDNA libraries with the RNA Library Prep Kit (Illumina, San Diego, CA, USA). The resulting libraries were sequenced on a HiSeq2000 platform (Illumina) to generate 100-bp paired-end reads.

### Processing of sequence data and mapping reads to reference genome

The sequenced data were filtered by removing adaptor sequences, empty reads, reads with more than 5% unknown nucleotides, low-quality sequences (base quality ≤ 20), or sequences with >10% Ns using SOAPnuke (version 1.5.2)^40^. Clean reads were mapped to the reference genome sequence using HISAT (version 0.1.6-beta)^41^. The reads were assembled into transcripts and compared with reference gene models using Cufflinks^42^. Gene expression was quantified using RSEM (RNA-Seq by Expectation Maximization, version 1.2.12)^43^. The data were normalised as fragments per kilobase of transcript per million fragments mapped (FPKM)^44^. The differences in transcript abundance between two genotypes were calculated based on the ratio of FPKM values. The FDR control method was used to identify the threshold of the *P* value using Cuffdiff (included in the cufflinks package). Only transcripts with *P* ≤ 0.001 and |log2 (TP/SP)| > 1 were further analysed.

### KEGG pathway enrichment analyses of DAPs and DEGs

The KEGG pathway enrichment analysis of the DAPs or DEGs was conducted using the KOBAS2.0 website (http://kobas.cbi.pku.edu.cn/)^45^. We identified significantly enriched metabolic pathways by using the hyper geometric test (http://en.wikipedia.org/wiki/Hypergeometric_distribution). The metabolic pathways that were significantly enriched compared with the whole genome background were considered with corrected values (*P* < 0.05).

### Quantitative real-time PCR analysis

Total RNA was used as the template for cDNA synthesis with reverse transcriptase (Takara Bio, Kusatsu, Japan) following the manufacturer’s instructions. The qRT-PCR analyses were conducted using the SYBR premix Ex Taq™ kit (Takara, Dalian, China) and a LightCycler^®^ 480 instrument (Roche, IN, USA), with gene-specific primers designed using Primer3 software^46^. The reactions were conducted in a 20-µl volume containing 10 µl 2 × SYBR Green Master Mix (Takara), 300 nM each primer (Supplementary Table S5), and 2 µl 10-fold diluted cDNA template. The qRT-PCR program was as follows: 95 °C for 10 s; and 40 cycles of 95 °C for 15 s and 60 °C for 30 s. The qRT-PCR analyses involved three biological replicates, each with two technical replicates. Relative gene expression levels were calculated according to the 2^−ΔΔCt^ method^47^.

### Data availability

The mass spectrometry proteomics data have been deposited to the ProteomeXchange Consortium via the PRIDE partner repository under the dataset identifier PXD009129.

## Electronic supplementary material

Supplementary Tables and Figures

Dataset 1

## References

[1] Yuan Z, Yin Y, Qu J, Zhu L, Li Y (2007). Population Genetic Diversity in Chinese Pomegranate (Punica granatum L.) Cultivars Revealed by Fluorescent-AFLP Markers. Journal of Genetics and Genomics.

[2] Levin GM (1994). Pomegranate (Punica granatum L.) plant genetic resources in Turkmenistan. Plant Genetic Resources Newsletter.

[3] Lichanporn I, Srilaong V, Wongsaree C, Kanlayanarat S (2008). Effect of storage temperature on peel color and physiological changes of longkong fruit (Aglaia dookkoo Griff). Acta Horticulturae.

[4] Tao J, Zhang S, An X, Zhao Z (2003). Effects of light on carotenoid biosynthesis and color formation of citrus fruit peel. Chinese Journal of Applied Ecology.

[5] Tao J, Zhang S, Zhang L, An X, Liu C (2003). Relation ship Between Color Formation and Change in composition of Carotenoids in Peel of Citrus fruit. Acta Photophysiologica Sinica.

[6] Lado J (2015). Fruit shading enhances peel color, carotenes accumulation and chromoplast differentiation in red grapefruit. Physiol Plant.

[7] Liu T (2015). Improved peach peel color development by fruit bagging. Enhanced expression of anthocyanin biosynthetic and regulatory genes using white non-woven polypropylene as replacement for yellow paper. Scientia Horticulturae.

[8] Bai S (2017). Transcriptome analysis of bagging-treated red Chinese sand pear peels reveals light-responsive pathway functions in anthocyanin accumulation. Scientific reports.

[9] Honda C (2002). Anthocyanin biosynthetic genes are coordinately expressed during red coloration in apple skin. Plant Physiology & Biochemistry.

[10] Voelckel C, Gruenheit N, Lockhart P (2017). Evolutionary Transcriptomics and Proteomics: Insight into Plant Adaptation. Trends in plant science.

[11] Ono NN (2011). Exploring the Transcriptome Landscape of Pomegranate Fruit Peel for Natural Product Biosynthetic Gene and SSR Marker Discovery(F). Journal of integrative plant biology.

[12] Ross PL (2004). Multiplexed protein quantitation in Saccharomyces cerevisiae using amine-reactive isobaric tagging reagents. Molecular & Cellular Proteomics.

[13] Wu HX (2014). Transcriptome and proteomic analysis of mango (Mangifera indica Linn) fruits. Journal of proteomics.

[14] Guo X, Xu J, Cui X, Chen H, Qi H (2017). iTRAQ-based Protein Profiling and Fruit Quality Changes at Different Development Stages of Oriental Melon. BMC plant biology.

[15] Li L (2013). Quantitative proteomic investigation employing stable isotope labeling by peptide dimethylation on proteins of strawberry fruit at different ripening stages. Journal of proteomics.

[16] Zhou Y, Wu X, Zhang Z, Gao Z (2015). Comparative proteomic analysis of floral color variegation in peach. Biochemical and biophysical research communications.

[17] Li LQ (2017). Comparative Morphology, Transcription, and Proteomics Study Revealing the Key Molecular Mechanism of Camphor on the Potato Tuber Sprouting Effect. International journal of molecular sciences.

[18] Chen J (2017). iTRAQ and RNA-Seq Analyses Provide New Insights into Regulation Mechanism of Symbiotic Germination of Dendrobium officinale Seeds (Orchidaceae). Journal of proteome research.

[19] Pandey A, Mann M (2000). Proteomics to study genes and genomes. Nature.

[20] Muers M (2011). Gene expression: Transcriptome to proteome and back to genome. Nature Reviews Genetics.

[21] Ben-Simhon Z (2015). A “White” Anthocyanin-less Pomegranate (Punica granatum L.) Caused by an Insertion in the Coding Region of the Leucoanthocyanidin Dioxygenase (LDOX; ANS) Gene. Plos One.

[22] Tunen AJ, Mur LA, Recourt K, Gerats AG, Mol JN (1991). Regulation and manipulation of flavonoid gene expression in anthers of petunia: the molecular basis of the Po mutation. The Plant cell.

[23] Lin-Wang K (2010). An R2R3 MYB transcription factor associated with regulation of the anthocyanin biosynthetic pathway in Rosaceae. BMC plant biology.

[24] Takos AM (2006). Light-induced expression of a MYB gene regulates anthocyanin biosynthesis in red apples. Plant physiology.

[25] Tsuda T, Yamaguchi M, Honda C, Moriguchi T (2004). Expression of anthocyanin biosynthesis genes in the skin of peach and nectarine fruit. Journal of the American Society for Horticultural Science American Society for Horticultural Science.

[26] Li SJ, Deng XM, Mao HZ, Hong Y (2005). Enhanced anthocyanin synthesis in foliage plant Caladium bicolor. Plant Cell Reports.

[27] Liu XJ (2012). Methylation effect on chalcone synthase gene expression determines anthocyanin pigmentation in floral tissues of two Oncidium orchid cultivars. Planta.

[28] Wei H (2015). Comparative Transcriptome Analysis of Genes Involved in Anthocyanin Biosynthesis in the Red and Yellow Fruits of Sweet Cherry (Prunus avium L.). Plos One.

[29] Lakshminarayana S, Mathew AG (2010). Leucoanthocyanidins of Sapola Fruit. Journal of Food Science.

[30] Quattrocchio F, Verweij W, Koes R (2005). Flavonoids: a colorful model for the regulation and evolution of biochemical pathways. Trends in Plant Science.

[31] Grotewold E (2006). The genetics and biochemistry of floral pigments. Annual Review of Plant Biology.

[32] Ogata J, Kanno Y, Itoh Y, Tsugawa H, Suzuki M (2005). Anthocyanin biosynthesis in roses. Nature.

[33] Thygesen PW, Dry IB, Robinson SP (1995). Polyphenol oxidase in potato. A multigene family that exhibits differential expression patterns. Plant Physiology.

[34] Anderson NA (2015). Manipulation of guaiacyl and syringyl monomer biosynthesis in an arabidopsis cinnamyl alcohol dehydrogenase mutant results in atypical lignin biosynthesis and modified cell wall structure. Plant Cell.

[35] Hu M, Qiu Z, Zhou P, Xu L, Zhang J (2012). Proteomic analysis of ‘Zaosu’ pear (Pyrus bretschneideri Rehd.) and its red skin bud mutation. Proteome Science.

[36] Ben-Simhon Z (2011). A pomegranate (Punica granatum L.) WD40-repeat gene is a functional homologue of Arabidopsis TTG1 and is involved in the regulation of anthocyanin biosynthesis during pomegranate fruit development. Planta.

[37] Unwin RD, Griffiths JR, Whetton AD (2010). Simultaneous analysis of relative protein expression levels across multiple samples using iTRAQ isobaric tags with 2D nano LC-MS/MS. Nature Protocols.

[38] Wen B (2014). IQuant: an automated pipeline for quantitative proteomics based upon isobaric tags. Proteomics.

[39] Savitski MM, Wilhelm M, Hahne H, Kuster B, Bantscheff M (2015). A Scalable Approach for Protein False Discovery Rate Estimation in Large Proteomic Data Sets. Molecular & Cellular Proteomics Mcp.

[40] Li R, Li Y, Kristiansen K, Wang J (2008). SOAP: short oligonucleotide alignment program. Bioinformatics.

[41] Kim D, Langmead B, Salzberg SL (2015). HISAT: a fast spliced aligner with low memory requirements. Nature Methods.

[42] Trapnell C (2012). Differential gene and transcript expression analysis of RNA-seq experiments with TopHat and Cufflinks. Nature Protocols.

[43] Li B, Dewey CN (2011). RSEM: accurate transcript quantification from RNA-Seq data with or without a reference genome. BMC Bioinformatics.

[44] Mortazavi A, Williams BA, Mccue K, Schaeffer L, Wold B (2008). Mapping and quantifying mammalian transcriptomes by RNA-Seq. Nature Methods.

[45] Kanehisa FM, Tanabe M, Sato Y, Morishima K (2017). KEGG: new perspectives on genomes, pathways, diseases and drugs. Nucleic Acids Res..

[46] Koressaar T, Remm M (2007). Enhancements and modifications of primer design program Primer3. Bioinformatics (Oxford, England).

[47] Livak KJ, Schmittgen TD (2001). Analysis of relative gene expression data using real-time quantitative PCR and the 2(-Delta Delta C(T)) Method. Methods.

