# Supplementary material for: Complementary iTRAQ-based proteomic and RNA sequencing-based transcriptomic analyses reveal a complex network regulating pomegranate (Punica granatum L.) fruit peel colour
Source: Sci Rep. 2018 Aug 17;8:12362. doi: 10.1038/s41598-018-30088-3 (PMC6098015; doi:10.1038/s41598-018-30088-3)
Supplement: Supplementary file 1 — Supplementary Tables and Figures [file 41598_2018_30088_MOESM1_ESM.pdf]

Complementary iTRAQ-based proteomic and RNA sequencing-based transcriptomic analyses reveal a complex network regulating pomegranate (*Punica granatum* L.) fruit peel colour

Xiang Luo[*], Da Cao[*], Haoxian Li[*], Diguang Zhao, Hui Xue, Juan Niu, Lina Chen, Fuhong Zhang & Shangyin Cao

Zhengzhou Fruit Research Institute, Chinese Academy of Agricultural Sciences, Zhengzhou 450009, P. R. China

[*]These authors contributed equally to this work.

·Correspondence and requests for materials should be addressed to S.C

E-mail: s.y.cao@163.com

Zhengzhou Fruit Research Institute, Chinese Academy of Agricultural Sciences, Zhengzhou 450009, P. R. China

Tel: +86-0371-65330990

Fax: +86-0371-65330963

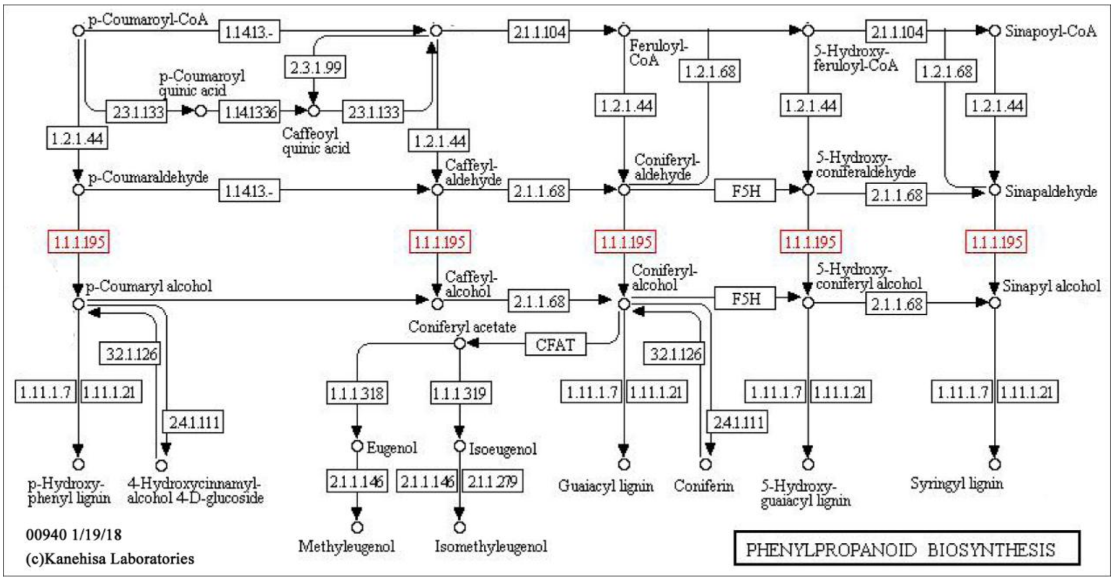

Supplementary Figure S1. Analysis of cinnamyl-alcohol dehydrogenase functions related to phenylpropanoid biosynthesis with KEGG developed by Kanehisa Laboratories[45].

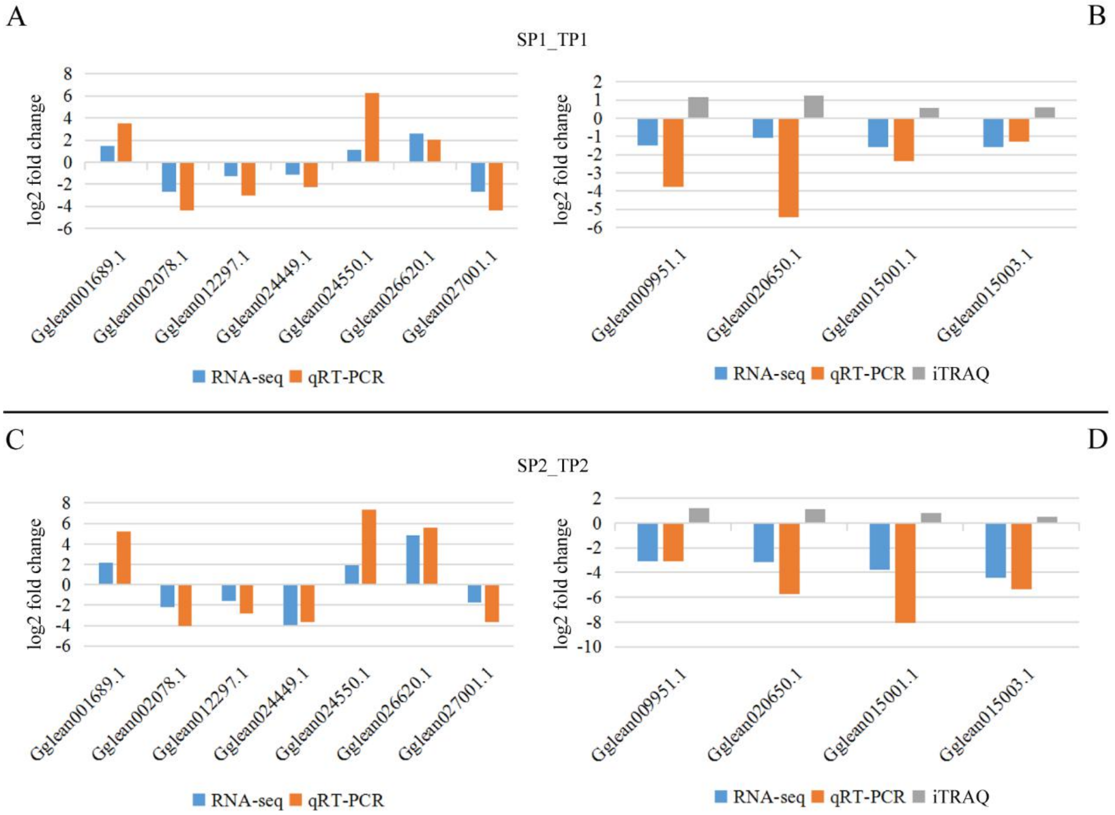

Supplementary Figure S2. qRT-PCR analysis of selected genes in (A and B) SP1_TP1 and (C and D) SP2_TP2.

Supplementary Table S1. Filtered iTRAQ and RNA sequencing data.

Sample	iTRAQ				RNA-seq			
	Total spectra	Unique Spetra	Unique Peptide	Protein	Total Raw Reads	Total Clean Reads	Total Mapping Ratio	Uniquely Mapping Ratio
SP1	237421	54346	18407	5357	64852984	37431229	45.23%	37.34%
SP2	237421	54346	18407	5357	61986029	35631903	44.06%	36.22%
TP1	237421	54346	18407	5357	59988046	34677616	42.25%	34.53%
TP2	237421	54346	18407	5357	67458765	38763711	41.89%	33.33%

Supplementary Table S3. Common DAPs among the four candidate pathways.

Pathway	Common DEPs
Anthocyanin biosynthesis	Gglean022014.1
	Gglean030887.1
Stilbenoid, diarylheptanoid and gingerol biosynthesis	Gglean020115.1
	Gglean017092.1
	Gglean017092.1
	Gglean017092.1
	Gglean017162.1
	Gglean022299.1
	Gglean030413.1
Flavonoid biosynthesis	Gglean021885.1
	Gglean002595.1
	Gglean024933.1
	Gglean000166.1
	Gglean024550.1
	Gglean024451.1
	Gglean009951.1
	Gglean002270.1
	Gglean020650.1
Phenylpropanoid biosynthesis	Gglean023090.1
	Gglean015001.1
	Gglean030344.1
	Gglean004667.1
	Gglean015223.1
	Gglean005438.1
	Gglean015003.1
	Gglean002739.1
	Gglean026319.1
	Gglean006509.1
	Gglean008923.1
	Gglean004749.1
	Gglean015152.1
	Gglean017305.1
	Gglean025624.1
	Gglean026354.1

Supplementary Table S4. Common DEGs among the four candidate pathways.

Pathway	Common DEGs
Anthocyanin biosynthesis	*Gglean024784.1*
	Gglean011259.1
	Gglean024783.1
	Gglean019130.1

	Gglean021842.1
	Gglean030299.1
	Gglean017094.1
	Gglean019885.1
	Gglean017523.1
	Gglean022291.1
	Gglean025205.1
	Gglean019884.1
	Gglean015246.1
	Gglean015968.1
Stilbenoid, diarylheptanoid and gingerol biosynthesis	*Gglean018889.1*
	Gglean015686.1
	Gglean017252.1
	Gglean020272.1
	Gglean027026.1
	Gglean004433.1
	Gglean016885.1
	Gglean024512.1
	Gglean022304.1
	Gglean000381.1
	Gglean020112.1
	Gglean013382.1
	Gglean015692.1
	Gglean026629.1
	Gglean009951.1
	Gglean020650.1
	Gglean012297.1
	Gglean024550.1
	Gglean024449.1
	Gglean025015.1
	Gglean001689.1
	Gglean026620.1
	Gglean002570.1
	Gglean002078.1
	Gglean006664.1
	Gglean027001.1
	Gglean000166.1
Flavonoid biosynthesis	*Gglean002629.1*
	Gglean018019.1
	Gglean014037.1
	Gglean015001.1
	Gglean015003.1
Phenylpropanoid biosynthesis	*Gglean026937.1*
	Gglean007009.1

Gglean004745.1
Gglean008923.1
Gglean019078.1
Gglean018745.1
Gglean014679.1
Gglean016279.1
Gglean025214.1
Gglean002744.1
Gglean008959.1
Gglean004943.1
Gglean004996.1
Gglean028708.1
Gglean031223.1
Gglean015153.1
Gglean010746.1
Gglean004579.1
Gglean031042.1
Gglean027772.1
Gglean008643.1
Gglean013008.1
Gglean022575.1
Gglean028815.1
Gglean024774.1
Gglean018674.1
Gglean007582.1
Gglean021760.1
Gglean026132.1
Gglean026319.1
Gglean003999.1
Gglean004749.1
Gglean007149.1
Gglean016128.1
Gglean028143.1
Gglean025275.1
Gglean019365.1
Gglean016790.1
Gglean017886.1
Gglean011165.1
Gglean022649.1
Gglean026442.1
Gglean013351.1
Gglean005187.1
Gglean004396.1
Gglean026874.1

	Gglean004746.1
	Gglean003647.1
	Gglean029542.1
	Gglean019513.1
	Gglean010569.1
	Gglean029864.1
	Gglean029861.1
	Gglean023909.1

Supplementary Table S5. Primers of mRNAs used in this study.

Primer ID	Forward primers (5'to3')	Reverse primer (5'to3')
Gglean001689.1	ACTACACAATGGTCGAGCCG	CATGAAGTTGCTGCGCTTCC
Gglean002078.1	CAATGCACAGGGCAATCACG	TTGACCGTCTTGTAGAGCCC
Gglean009951.1	CTACCGGACTCTGATGGTGC	ACATGTTCCCGTACTCGCTC
Gglean012297.1	ATCTTCTGGATCGCACACCC	AGGATGAAGAGGACGCAAGC
Gglean015001.1	CAGCCCACCTATAATGGCGT	GCCTTACCGAATTTGACCGC
Gglean015003.1	ATATCGAGACGGTCACCCCT	AGCTTAGATGTCTTGCCCGC
Gglean020650.1	GGCCATAGTCTACCCCCTCA	CGCTAGCTCGAGGTCCTTAC
Gglean024449.1	TTGGAGGACATGTTCGTCGG	TTGCGGCCATTAACGAGTTC
Gglean024550.1	GCAACCCGAAGGCAATCTTC	ATGTTCTTTGGAGGCCGGAG
Gglean026620.1	CGAGATCCTCAGCAACGGAA	TCCTGAAGAGCTTGTGCTCG
Gglean027001.1	TGCCCAAGTCAATCGGCTAC	TCCCAATCGAAGGAGTGCAG
